# Regarding the Amazing Choreography of Clathrin Coats

**DOI:** 10.1371/journal.pbio.1001037

**Published:** 2011-03-22

**Authors:** Linton M. Traub

**Affiliations:** Department of Cell Biology and Physiology, University of Pittsburgh School of Medicine, Pittsburgh, Pennsylvania, United States of America

The growth of contemporary cell biology is due in large part to technological advances. In the 1950s, electron micrographs of thin sections first provided unrivaled in situ views of the delicate intracellular architecture and fine structure of organelles, whereas new subcellular fractionation methods gave access to various biochemical components—especially proteins—enriched in different cellular fractions. A central tenet that emerged from these pioneering studies is that the intracellular biosynthetic and endocytic membrane systems of eukaryotic cells are functionally interconnected, and exchange of material between them often occurs in small (50–100 nm diameter), roughly spherical membranous transport vesicles. In electron micrographs, these vesicles are typically seen in close proximity to a membrane compartment and are frequently covered on their cytosolic face with a fuzzy proteinaceous coating. Subsequent technical advances facilitated further discovery and progress: genetic screens in model organisms and refinement of subcellular fractionation to facilitate cell-free reconstitution of transport reactions allowed the identification and purification of key regulatory and structural components. Persuasively, many of the genes discovered in genetic screens encoded the proteins purified biochemically. More recently, genome sequencing and proteomics efforts have bolstered the identification of sorting components, leading to long lists of evolutionarily conserved proteins that are involved in specific sorting operations at different membranes.

## Coat Proteins

**Figure 1:**
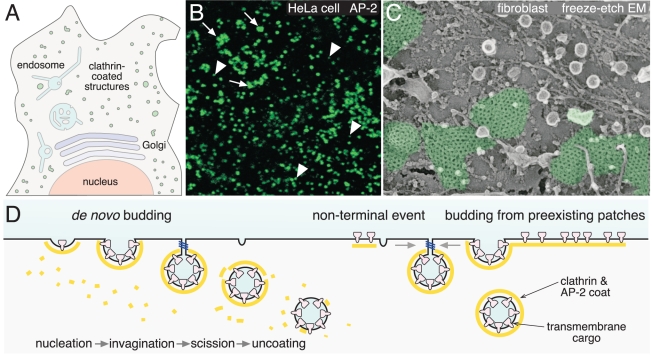
Clathrin-mediated endocytosis. (A) A schematic bird's-eye view of a mammalian cell showing randomly scattered clathrin-coated structures (green) positioned on the adherent cell surface. (B) Confocal immunofluorescence image of the adherent surface of HeLa cells stained with an antibody against the AP-2 adaptor protein showing coexistence of diffraction-limited spots (arrowheads) and large clathrin patches (arrows). (C) High-resolution electron micrograph of the adherent surface of a cultured fibroblast (courtesy of John Heuser) showing areas of flat clathrin lattice (pseudocolored in green). (D) Schematic depiction of the process of clathrin-coated vesicle assembly at the various types of bud site analyzed by Taylor et al. [33].

**Figure 2:**
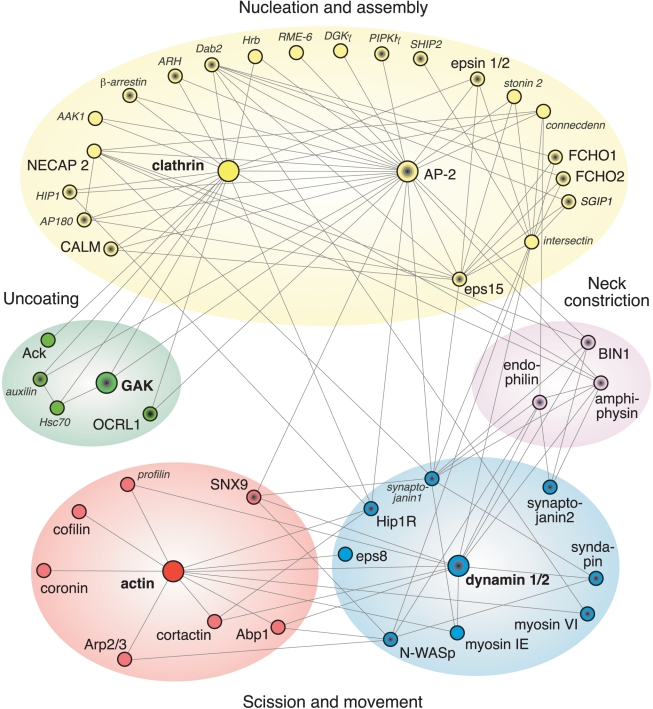
The vertebrate endocytic clathrin-coat protein interaction network. Hub-and-spoke depiction of a selected subset of the known proteins participating in clathrin-mediated endocytosis. Established interactions are indicated by the spokes. Modules are colored as in Taylor et al. [33] and the proteins they analyzed are shown in larger font. Note that not all of the temporally defined modules are shown here. The symbols with black centers indicate proteins that bind to phosphatidylinositol 4,5-bisphosphate, a lipid marker of the plasma membrane. How can clathrin and AP-2 each bind to so many partners (at once)? The functional clathrin molecule has at least 15 physically separate interaction surfaces while each AP-2 complex has over ten.

Clathrin-mediated endocytosis is the archetype of a vesicular transport reaction that sorts specific cargo for transportation to another intracellular compartment (in this case, endosomes) [1]. This process is conserved from unicellular eukaryotes, like yeast, to plants and mammals. It entails the selective retention of certain membrane proteins within a progressively dimpling region of the plasma membrane, coated on the cytosolic side with a polymerized lattice of the protein clathrin [2] (Figure 1). The clathrin envelops the plasma membrane region into a small vesicle that buds off into the cell, carrying with it the selected cargo. Early models of this process revolved around a triad of molecular components that are, alas, still common in textbook-type schematic renditions of the process [3]. This core triad comprises an inner layer of various transmembrane proteins (and their attached extracellular ligands)—the cargo—and the structural outer clathrin layer that deforms the membrane into a vesicle, bridged by an intervening layer of selectivity-determining adaptors, principally adaptor protein 2 (AP-2). There is no doubt these constituents are critical; disruption of genes encoding AP-2 or clathrin is, typically, lethal [4]. Yet, what we have learned over the past decade is that the assembly of these core components is augmented and precisely regulated at vesicle bud sites by an abundance of additional proteins (Figure 2).

At least 40 different proteins participate in the construction of a clathrin-coated endocytic vesicle [1],[5],[6]. Precisely when and how the many distinct proteins interact as the vesicle forms, how information is relayed, and how directionality is assured without malfunction, given there is no obvious coupled input of energy to instigate budding, is currently uncertain. Also, it remains possible that various combinations of these many factors might build structurally distinct sorting structures, perhaps associated with separable functions [7]–[10]. Some empirical evidence for this actually exists: electron micrographs show both isolated ∼100-nm diameter clathrin-coated buds as well as large expanses of apparently planar clathrin lattice at the surface of various cell types [11]–[16].

## Questions and Controversy

Past insights into clathrin-mediated endocytosis have come mainly from studies of chemically fixed or ground-up cells [17]. The latest wave of discovery—again, based on new technology—uses sophisticated live-cell imaging to understand coat assembly over the minute or two that it takes the cell to create a new vesicle. By using fluorescently tagged clathrin or AP-2, researchers have confirmed the existence of variably sized patches, at least in most cell types [18]–[22]. Furthermore, time-resolved imaging reveals that the small clathrin buds (seen as ≤200-nm diameter diffraction-limited spots) and large clathrin patches can have different lifetimes [19],[23],[24]. This morphological and temporal plasticity raises several fundamental questions, not the least of which is, what is the physiological significance of the coexistence of the small clathrin spots and the longer-lived and rather sessile, large, flat clathrin patches? In the baker's yeast *Saccharomyces cerevisiae*, assembled clathrin caps the ends of regularly sized, bullet-shaped invaginations of plasma membrane, termed cortical actin patches, which are involved in endocytic uptake [25]. Here, assembly of branched actin microfilaments is inextricably intertwined with the proper operation of these vesicular shuttles, and elegant live-cell imaging has cataloged the temporal behavior of numerous endocytic proteins, in the process defining a cascade of operational modules [26]. So, although originally appearing quite dissimilar to clathrin-mediated uptake in complex eukaryotes, it is now clear that there are many orthologous components and mechanistic parallels between this process in *S. cerevisae* [6],[26], another distantly related yeast, *Schizosaccharomyces pombe* [27], and more complex organisms [28]. Nevertheless, there is currently a contentious dialogue over whether actin is (similarly) required for routine clathrin-dependent endocytosis in metazoans [19],[23],[29], partly because whereas in *S. pombe* actin outnumbers clathrin by >100-fold in cortical patches [27] in vertebrate cells, massed microfilaments are not routinely seen at clathrin-coated buds. Also still in dispute is what the different sized and lived clathrin-coated structures actually are and might do, considering that yeast endocytic buds have a relatively constant size and a different geometry to metazoan clathrin-coated buds [25]. Some evidence points to actin being required only at large clathrin-coated areas [19], or when bulky cargo material is being imported into vertebrate cells [30].

## Budding Goes Live

To deal with these confounding issues, investigators in the field have pursued two directions. One is to define carefully the temporal parameters of clathrin coat assembly in an atypical mammalian cell line (BS-C-1), chosen for the inherent uniformity of the size and behavior of the clathrin structures on the surface of these cells [9],[31],[32]. The other is to try, in an unbiased way, to decipher systematically the functional relationship (if any) between the various sized and lived clathrin structures in cultured cells [19],[23]. In this issue of *PLoS Biology*, Merrifield and colleagues present a tour de force analysis of the latter type [33]. Their approach uses total internal reflection fluorescence microscopy (TIR-FM), an optical technique that uses an exponentially decaying evanescent field to visualize fluorescent molecules within ∼200 nm of the glass coverslip, i.e., at and just below the cell surface. It hinges on a clever twist: using a pH-sensitive variant of green fluorescent protein (called pHlourin) fused to the extracellular portion of a generic cargo molecule, the transferrin receptor. By expressing this pHlourin–transferrin receptor hybrid in NIH-3T3 fibroblasts and rapidly oscillating the pH of the bathing medium between pH 5 and pH 7, Taylor et al. use TIR-FM to discriminate between receptors on the cell surface and those that have just entered the cell (now protected from fluorescence quenching at pH 5). By simultaneously expressing a red fluorescently tagged clathrin protein, the relative clathrin and transferrin receptor dynamics can be unraveled with two-color TIR-FM.

To begin with, the pHlourin–transferrin receptor accumulates at both diffraction-limited spots and large patches [33], so the surface reporter enters both types of clathrin structure apparently at random. Also, the intensity of the transferrin receptor reporter signal at pH 7 is proportional to the clathrin signal. The crucial question, however, is whether the large plaques that accumulate the receptor are also competent to internalize it [19]. What is interesting is that there is no correlation between the amount of transferrin receptor internalized and the overall size of the surface clathrin (or pH 7 pHlourin) signal it is initially coincident with [33]; however, at pH 5—looking at just-internalized vesicles—the pHlourin–transferrin receptor signals are remarkably consistent and do not correlate with the size of the “host” region. This argues that although buds can form at various-sized clathrin lattice zones, the individual clathrin-coated vesicles that emanate from any of these zones have approximately the same packaging capacity. The implication is that coated vesicles of relatively uniform size can bud from larger, even flat clathrin lattices. Consistent with this, in typical cultured cells, most clathrin-coated vesicles at the plasma membrane are ∼100 nm in diameter, irrespective of the size of the coated patch from which they just originated. The data are also nicely concordant with local fluctuations in cargo or coat fluorescence seen at larger clathrin patches [20],[22], indicative of budding of sub-regions of expansive clathrin assemblies.

## The Whole Kit and Caboodle

By determining precisely, to within two seconds, when a cargo-laden vesicle detaches, Taylor et al. have produced a functionally defined, unmistakable temporal landmark for vesicle scission. This differs importantly from simply following the abrupt disappearance of the clathrin signal from the TIR-FM illumination field, which could also be due to (later) vesicle uncoating or physical movement out of the evanescent field. Moreover, upon budding not all clathrin-positive structures lose completely their surface-apposed clathrin: there are also budding events in which a small residue of the coat remains at the bud site to nucleate a second round of vesicle assembly within ∼40 seconds [19],[21],[33].

Dynamin is a large GTPase that plays an essential role in vesicle release from the plasma membrane. By expressing a red fluorescently tagged dynamin protein instead of the clathrin, and again using two-color TIR-FM, Taylor et al. show that dynamin appears immediately preceding the occurrence of a pH 5-stable pHlourin–transferrin receptor signal, nicely validating the assay. Accurately pinpointing the moment of scission also provides the opportunity to catalog other participants in the process; so, with the two-color TIR-FM assay in hand, the Merrifield group turned their attention to 33 other proteins. What they learned is instructive and far-reaching. Cluster analysis of kinetic profiles of the many proteins examined defines seven operational modules on the basis of their similar dynamics [33], highlighting the similarity with yeast [28]. The temporal order of the modules is stereotyped with respect to the budding and scission process, but the order of protein appearance and buildup within each module is not identical. As one might expect, the amount of early arriving “pioneer” components (in the earliest clathrin module) at a given bud site correlates with the size of the bud zone and also with its lifetime. The amount of the later-acting proteins and actin machinery, however, does not. This reinforces the notion that the coat machinery makes individual vesicles carrying roughly quantum-sized loads of cargo.

Messenger RNA analysis confirms that the bulk of the proteins followed by Taylor et al. are endogenously expressed in NIH-3T3 cells and the transfected, fluorescently tagged versions are detectable in the majority of clathrin-coated structures [33]. This puts to rest the parsimonious assertion that the complexity of clathrin coat assembly is wildly overstated and that tissue-specific expression patterns dramatically limit the connectivity of the endocytic protein interaction web (Figure 2). Also, there is no evidence from this study that the large and small clathrin patches have fundamentally different protein compositions, suggesting that, in these cells at least, there are no distinct pools of clathrin-coated structures with different protein compositions at steady state. The actin and dynamin profiles overlap, although that of actin is broader: actin typically begins to assemble ∼20 seconds before dynamin. This argues that actin indeed operates at all clathrin bud sites on the surface. The observation that certain proteins, particularly those from the late-acting modules, are not invariably found at each bud site is probably due to TIR-FM detection limits and the fact that there is always an endogenous, unlabeled pool of protein in all of these transient transfection experiments. With this in mind, it is worthwhile noting that, unfortunately, this setup does not allow estimation of the stoichiometry of the various components, as is possible in yeast [27].

If there is indeed only one basic mode of clathrin vesicle budding, it still leaves open why sometimes these arise de novo and sometimes from preexisting larger clathrin assemblies (Figure 1). Perhaps this relates to the way that bud sites are initially nucleated? What cue makes a residue of a coat linger at a bud site or enlarge to generate a persistent staging area for numerous subsequent rounds of import? And, curiously, why do the planar regions not curve? Recent evidence shows that clathrin bud sites expand and endure longer with strong cell adhesion to the substrate [23], so physical forces may be at work. Also, despite the kinetic elegance of the work, we still do not really understand what heralds either the early or later arriving proteins. The microscopic events cataloged are, in fact, only manifestations of physical interactions between various proteins and lipids (Figure 2). How the precise timing of protein entry and exit involves specific binding sites and is affected by occupancy, stoichiometry, and possibly post-translational modifications of the partner proteins will be important to understand. Still, the rich dataset presented will undoubtedly help researchers to produce predictive computational models of the process, which are sorely needed to allow further dissection and understanding of this important and intricate, but brief, vesicle assembly activity.

## Abbreviations

AP-2: adaptor protein 2
TIR-FM: total internal reflection fluorescence microscopy
